# Level of medication self-management capacity among patients on ambulatory care exiting hospital pharmacy at Muhimbili National Hospital, Tanzania: a descriptive cross-sectional study

**DOI:** 10.1186/s13104-019-4772-4

**Published:** 2019-11-07

**Authors:** Hamu Mlyuka, Hija Salehe, Wigilya Mikomangwa, Manase Kilonzi, Alphonce Marealle, Ritah Mutagonda, George M. Bwire

**Affiliations:** 1 Clinical Pharmacy and Pharmacology Department, School of Pharmacy, Muhimbili University of Health and Allied Sciences, P.O. Box 65013, Dar es Salaam, Tanzania; 2 Department of Pharmaceutical Microbiology, Muhimbili University of Health and Allied Sciences, P.O. Box 65013, Dar es Salaam, Tanzania

**Keywords:** Medication self-management, Medication management capacity, Ambulatory care

## Abstract

**Objectives:**

Medication management capacity of a patient on ambulatory care is direct related to adherence. To our knowledge data on medication management capacity for ambulatory care patients exiting outpatient pharmacy outlets in Tanzania are scarce. This study aimed to determine the level of medication management capacity among patients on ambulatory care exiting Muhimbili National Hospital outpatient pharmacy outlet.

**Results:**

A total of 424 patients on ambulatory care participated in the study. Three hundred eighty-seven (91.3%) out of 424 interview questionnaires had complete data and qualified for data analysis. Majority (62.3%) out of 387 study participants had poor medication management capacity; 65.3% out of 387 patients were unable to correctly read the prescription and match the drugs they are carrying. More than half (57.4%) out of 387 participants were unable to correctly take the dose, 73.9% out of 387 were unable to correctly tell the dosing frequency and duration. Only 10.6% out 155 patients with prescription containing drugs with warning or precaution or contraindication or potential side effects were aware.

## Introduction

Adherence to medication is a key determinant of regimen effectiveness and safety. It is also a mediator of medical practices and patients’ outcomes [1]. Almost 20–50% of patients do not take their medications as prescribed and the situation is even worse in chronic condition [2]. In USA non-adherence causes 125,000 death and 10% hospital admission annually. Enormous healthcare financial burden up to US$ 289 billion per anum are attributed by medication non adherence [3].

Adherence to medication is direct related to medication management capacity (MMC) of an individual patient [4, 5]. MMC is defined as ‘‘the cognitive and functional ability to self-administer a medication regimen as it has been prescribed” [6]. Functional skills like correctly identifying the medication, opening the container, taking the right dose and timing the use of medication are components of medication self management. In an emphasis of this relationship Bailey and colleagues developed “medication self-management model” which aims to deconstruct the tasks involved in taking medication [7]. Medication management capacity tell us the accuracy of using medication while adherence based on pill count, refill or self-report provide an insight of how often one is taking the medication. The intervention focusing on boosting patients MMC have resulted into reduction in emergency department visiting and hospital admission [8].

MMC can be measured using Drug Regimen Unassisted Grading Scale (DRUGS) or Medication Management Ability Assessment (MMAA) [6, 9]. DRUGS is used to measure MMC by considering the four steps which are identification of the right medication, accessing by opening the container, picking the correct number/amount of tablet or syrup and providing elaborative timing of using the medication [6].

So far, in Tanzania the data on level of medication management are dearth. This study aimed to assess the level of medication management capacity among clients exiting hospital pharmacy outlets at Muhimbili National Hospital, Dar es Salaam Tanzania.

## Main text

### Methods

#### Study area and design

This was a descriptive cross-sectional study conducted from February to July, 2018 at Muhimbili National Hospital (MNH) outpatient pharmacy outlets. MNH is a national referral tertiary level research center and university teaching hospital with 1500 beds facility, attending 1000 to 1200 outpatients per day. The hospital is well equipped with laboratories, modern diagnostic facilities, specialized clinics as well as highly skilled manpower in all cadres of healthcare providers.

#### Study population and sample size

To be included in this study participant was supposed to be; (1) from outpatient clinic in MNH (2) carrying a prescription with more than one drug for oral use (3) carrying medicines he/she has received from pharmaceutical personnel at MNH outpatient pharmacy outlets (4) not blind (5) not deaf (6) not drunk (7) not a guardian (8) with a regimen of more than three days of use. Owing to scarcity of data on prevalence of level of medication management capacity (MMC) the 50% proportion was set as a reference population proportion to calculate the sample size. At the Z-score of 1.96 and margin of error of 5% the sample size was 385. To consider for partial filled or inconsistency 10% of initial sample was added to make 424.

#### Sampling technique

Consecutive sampling was employed to recruit study participants. The researcher stayed at the exit point of the outpatient pharmacy building. When the client was about to get outside the pharmacy building the researcher requested him/her for a brief discussion. If the client agreed, the purpose of the study was explained, and then he/she signed freely obtained consent form. Assessment for inclusion and exclusion criteria was done and those who fulfilled requirements were asked to continue with the interview.

#### Data collection

Face-to-face interview using semi-structured questionnaire was used to gather data for fulfilling this study objectives. The questionnaire consisted questions and directives to assess medication self management capacity. This was a minor modified adoption of DRUGS tool which was used by Kripalani and colleague [6] in assessing MMC. On medication identification part; patient was asked to read what has been written on the prescription, show the particular drug, tell about warning, precaution or contraindication, important possible side effects and interactions with food or other drugs on the prescription. The researcher used Medscape interaction checker online software to check for potential life threatening interaction and match with the participant’s response. The patient was then asked to open the identified drug and the researcher observed the process of opening. After opening the container, the patient was asked to tell the number of tablet(s) or amount of syrup/suspension/solution which he/she is suppose to be taking per dose. Finally the patient was asked to tell the researcher about intervals of time to take the next dose and the total duration he/she will be using the medication. The steps were repeated for each of the medication on the prescription. For each question, the overall response was correct only if the participant has managed to perform a particular task correctly for all the prescribed medications on the prescription. Modified Bloom cut off point from study by Abdullah and colleagues [10] was used to categorize the MMC of the participants. The MMC was rated as poor/inadequate (score < 50%), moderate (score: 50–59%), good (score: 60–69%), very good (70–79%) and excellent (80–100%).

#### Data analysis

Analysis was done using statistical package for social sciences (SPSS) version 23. Categorical data like gender, age groups, levels of education, level of MMC were summarized using frequency distribution and bar charts.

### Results

A total of 424 patients on ambulatory care participated in this study. Three hundred eighty-seven (91.3%) questionnaires out of 424 were properly filled and qualified for data analysis. Patient characteristics are summarized on Table 1. Majority of the study participants (66.4%) were women. Many participants aged between 30 and 49 years and more than half attained secondary level of education.

**Table 1:** Patients socio-demographic characteristics

Characteristics (N = 387)	n (%)
Gender	
Male	130 (33.6)
Female	257 (66.4)
Age, years	
20–29	32 (8.30)
30–39	133 (34.4)
40–49	145 (37.5)
50–59	50 (12.9)
> 60	27 (7)
Marital status	
Single	66 (17.1)
Married	218 (56.3)
Divorced	21 (5.4)
Widowed	19 (4.9)
Cohabit	63 (16.3)
Education level	
Never	15 (3.9)
Primary	88 (22.7)
Secondary	208 (53.7)
Tertiary	76 (19.6)

#### Patients’ medication management capacity

**Fig. 1:**
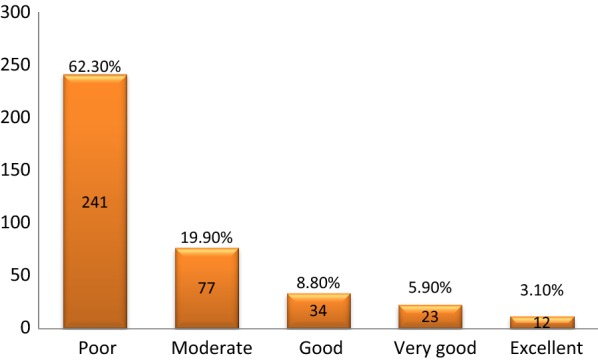
Bar chart of prevalence of medication management capacity

The overall grading showed that 241 (62.3%) of 387 study participants had poor MMC (Fig. 1). Majority of these participants were not able to identify, take the right dose or timing the frequency and duration as detailed below.

Table 2 shows the response to questions and activities to measure MMC. More than half of study participants (65.3%) were not able to correctly read and interpret the instructions written on their prescriptions and match with the medications on hand. All patients demonstrated an ability to open a container and take the medication. When asked to count pills or measure the mockup amount as directed on the prescription 222 (57.4%) were not able to correctly take the prescribed dose. When asked to explain the interval of hours to take the next dose and duration for each of the prescribed medications only 26.1% out of 387 participants were able to explain correctly.

**Table 2:** Response to attributes of measuring medication self management

Patient’s responses on medication self management questions or activities (N = 387)	
Item question or activity	Proportion of response (%)
1. Can you read the prescription of your medications and match the drug? (Able if read correctly for every drug on the Rx)	
Able	34.7
Unable	65.3
2. Show how to open the container of your medications	
Able	100
Unable	0
3. Show the number of tablets/capsule or amount of suspension/solution/syrup which you are suppose to take as prescribed (Correct if every drug number or amount is correct for all drugs on Rx)	
Correct	42.6
Incorrect	57.4
4. How many hours are supposed to elapse before you take another dose and how long will you take each drug? (Overall correct if the frequency and duration of every drug on the Rx is correctly explained)?	
Correct	26.1
Incorrect	73.9
5. Are you aware of potential side effects as result of using medications?	
Aware	54
Unaware	46
6. What is a possible potential side effect/warning/precaution due to your medication?^a^ (aware only if explained correctly for all drugs with possible side effects/warning/precaution) n = 155	
Aware	10.6
Unaware	89.4
7. What is your key source of medication information?	
Medical personnel	40
Internet	53
Others (bulletin, brochures, banners, etc.)	7
8. Are you aware of drug interaction?	
Aware	76
Unaware	24
9. Can you tell the possible dangerous interaction with regards to your medications?^b.^(able if explained possible interaction by indicating the drugs on the Rx) n = 60	
Able	7
Unable	93

^a^ The total number of prescription with potential side effects/warning/contraindication/precaution was 155 hence denominator for the proportions of response to item

^b^ The total number of prescription with possible potential interaction was 60 hence the denominator for proportions of response to the item

### Discussion

This study found an alarming level of prevalence of poor medication self-management among patients on ambulatory care. Many patients had difficulties on reading and interpreting what has been written on the prescription matching with what they are carrying on the hand. The medication self-management model has six tasks i.e. fill, understands, organize, take, monitor and sustain [7]. The scope of this study was to measure the ability of patients to understand the prescription, organizing the plan to take medication as prescribed, ability to open a container and take right number or amount of the medications, patient’s ability to monitor for side effects or warnings or interactions and the ability to sustain taking medication by understanding timing and duration of the regimen. Understanding the regimen on the prescription, taking the correct number or amount of dose, and timing of doses and duration of the regimen are areas which troubled many participants in this study. These findings concurred with what was observed in India after Gupta and colleagues conducted a study on the impact of patient information leaflets on medication adherence. Many patients had difficulties in timing of taking medications and duration of the regimen [11]. It is possible that, poor understanding of what has been written on the prescription or narrated by the pharmaceutical personnel is the root cause of poor performance on many of the tasks as testified in the work of Wolf and colleagues [12]. Majority of study participants reported to be secondary level graduates. Therefore, tough the study didn’t focus on associated factors; it is most probable that education level of the study participants had a major influence of MMC as it was reported in study by Kripalani et al. [6] and in the study which was conducted in Ethiopia [13].

With regard to medication information seeking behavior, this study has shown that more than half of the patients were not eager to ask their healthcare provider issues concerning their medications. This finding second what was observed in study which was conducted in South Africa [14].Tough it was not in the scope of this study, the reason may be uncomfortable platform of hospital pharmacy facilities, long waiting queue, unfriendly and brief contact time. All these have impact on ability of the patient to comprehend with written and verbal communication which play big role in understanding the regimen [15].

In this study many patients were not aware of the side effects or warning label or contraindication or precaution or drug interactions for their medications on hand. This is in line with findings from review by Bailey and colleagues [7] and study conducted in India [11]. This increase the risk of medication errors and adverse drug reactions which may jeopardize adherence to prescribed regimen. Most probable low level of education among study participants and brief contact time with healthcare providers may be the possible reasons [16].

Majority of the participants had poor ability to pick the correct number or amount of medications. The key reason may still be the same a poor understanding of the prescription regimen means poor understanding of the downstream tasks [6, 7].

Owing to detrimental consequences of poor MMC different implementation researches have come out with solution to mitigate the problem. Whenever possible, pharmaceutical personnel should use the principle of universal assumption i.e. assuming all patients have poor MMC [17]. But to maximize resources allocation identification of patient with poor MMC is the first important step. Normally these patients prefer no company, fail to establish rapport, tend to just release the written papers for a caregiver to read and take actions, use initials instead of signature and have unique ways (e.g. color, symbols) of identifying pills [6, 17].

Therefore, pharmaceutical personnel should identify poor MMC patients and exercise clear health communication skill strategies using plain language and teach-back method [18]. Also, proper medications packaging, labeling and dispensing using reading materials and language or symbol understood by patient [19] have proved effective to counter limitation of MMC [20]. Furthermore, intensive counseling and close follow-up are very important to patients with any MMC limitation [21, 22]. Volunteered social support on emotional, medication information, health reminder and tangible support are very important to patients with poor MMC because of their reluctance to seek help [23]. Continual training of healthcare providers on awareness, sensitization and collaboration to address the impact of poor MMC is important [24].

## Conclusion

The level of MMC for majority of outpatients attending national tertiary level hospital is very low. Further study need to focus on level of MMC and associated factors.

## Limitations

Consecutive sampling posed the risk of selection bias. Confounders like English proficiency as a communication language barrier, differences in cognitive and health literacy levels of the study participants were not measured. Also, sampled participants were from tertiary level hospital, with diversity in diseases and severity plus treatment durations.

## Data Availability

The dataset generated and/or analyzed during this study are available from the corresponding author upon reasonable request.

## Abbreviations

MMC: medication management capacity
DRUGS: Drug Regimen Unassisted Grading Scale
MMAA: Medication Management Ability Assessment
MNH: Muhimbili National Hospital
Rx: prescription
